# Supplementary material for: The interactome of intact mitochondria by cross-linking mass spectrometry provides evidence for coexisting respiratory supercomplexes
Source: Mol Cell Proteomics. 2017 Dec 8;17(2):216–32. doi: 10.1074/mcp.RA117.000470 (PMC5795388; doi:10.1074/mcp.RA117.000470)
Supplement: Supplemental Data [file supp_RA117.000470_133922_0_supp_23978_fzffwf.zip › spectra_annotation/mito_DR_spectra_annotation/196-1-10-1-14-1.pdf]

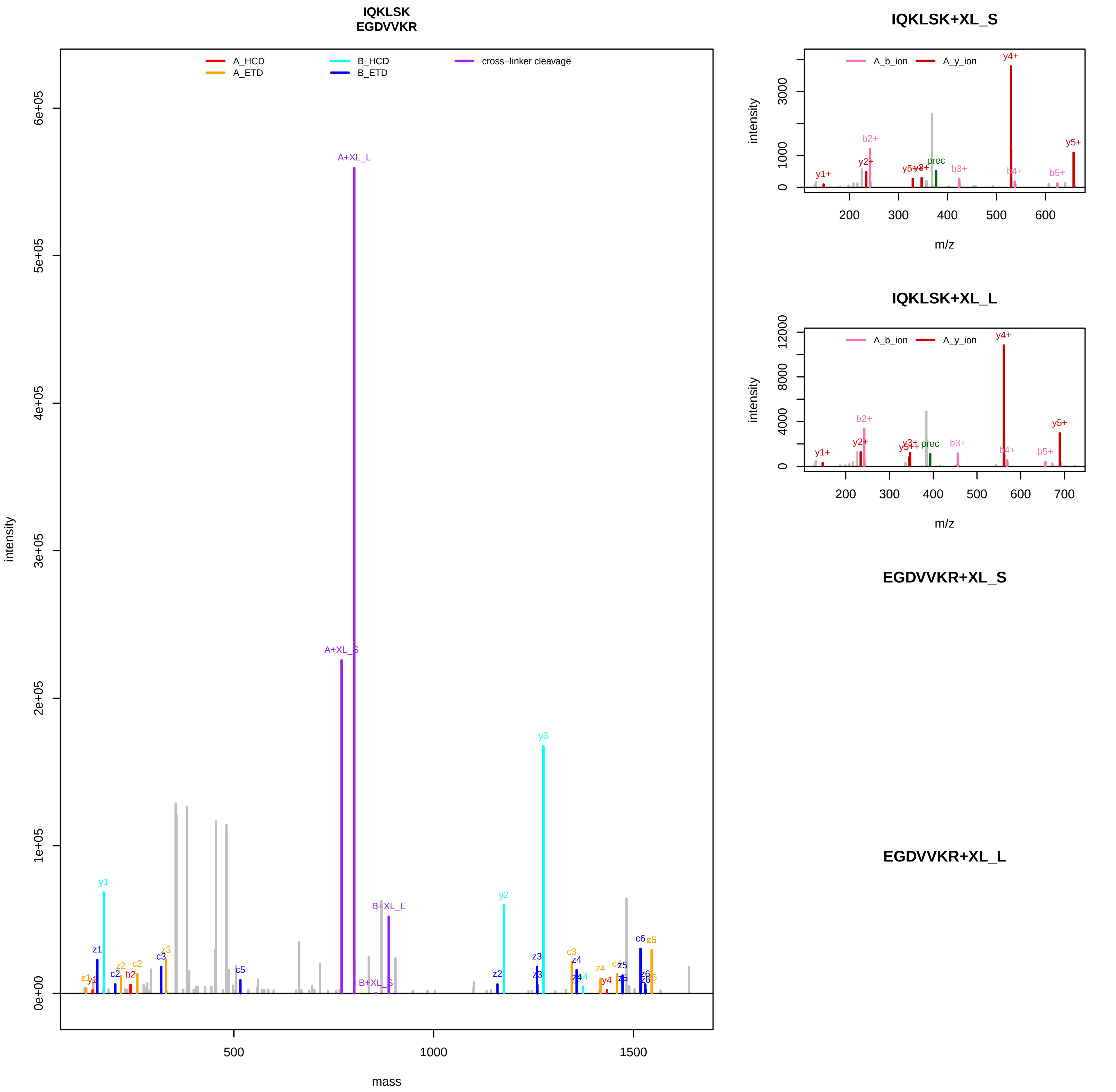

IQKLSK
EGDVVKR
A_HCD
A_ETD
B_HCD
B_ETD
cross−linker cleavage
intensity
0e+00
1e+05
2e+05
3e+05
4e+05
5e+05
6e+05
500
1000
1500
mass
A+XL_L
A+XL_S
B+XL_L
B+XL_S
y1
y2
y3
z1
z2
z3
z4
z5
z6
c2
c3
c4
c5
c6
b2
y4
IQKLSK+XL_S
A_b_ion
A_y_ion
intensity
0
1000
3000
200
300
400
500
600
m/z
y1+
y2+
b2+
y5++
y3+
prec
b3+
y4+
b4+
b5+
y5+
IQKLSK+XL_L
A_b_ion
A_y_ion
intensity
0
4000
8000
12000
200
300
400
500
600
700
m/z
y1+
y2+
b2+
y5++
y3+
prec
b3+
y4+
b4+
b5+
y5+
EGDVVKR+XL_S
EGDVVKR+XL_L
